# Putting the Brakes on Infrastructure? Judicial Review Challenges to HS2 and the Critique of ‘Litigant Power’

**DOI:** 10.1093/ojls/gqaf015

**Published:** 2025-05-19

**Authors:** Sam Guy

**Keywords:** judicial review, administrative law, infrastructure, planning, High-Speed 2, adversarial legalism

## Abstract

A growing critique regards judicial review as inhibiting infrastructure delivery on the basis of what I term ‘litigant power’, which may come to represent the dominant political critique of judicial review under the Labour administration. This differs from classic concerns of judicial power, focusing on how legal challenges by project opponents—notwithstanding their doctrinal outcome—can produce delay and embed a chilling overcaution among industry and policy makers. Having articulated the litigant power critique alongside judicial power, the article explores judicial review’s impacts on infrastructure delivery through a case study of the legal challenges to England’s High-Speed 2 railway project. I argue this litigation presents little evidence of judicial overreach, but in some ways supports litigant power concerns. Nevertheless, I suggest the litigant power critique risks oversimplification, especially in view of the radical reform often proposed, and it also downplays chilling effects associated with the constitution’s centralisation of government decision-making power.

## 1. Introduction

As discourse intensifies regarding Britain’s stagnant infrastructure delivery, public lawyers may have noted that judicial review reform is again reaching the political agenda. The previous Conservative government highlighted how judicial review claims (often enforcing consultation or environmental assessment requirements) can delay projects and create industry uncertainty.^1^ Meanwhile, within a wider emphasis on galvanising infrastructure delivery and house building through modifying planning, Sir Keir Starmer has indicated willingness to reform judicial review.^2^ After the previous government appointed Lord Banner KC to consider the case for reforming legal challenges to Development Consent Orders (DCOs) for Nationally Significant Infrastructure Projects under the Planning Act 2008,^3^ the Labour government has announced plans to implement some of Lord Banner’s recommendations.^4^ These political concerns resonate with a criticism beyond the infrastructure context that judicial review exerts a ‘chilling effect’ inhibiting executive policy making and implementation, which is increasingly being made by policy actors but is yet to attract public law scholars’ empirical attention.^5^

This article explores this theoretical criticism—that administrative law inhibits infrastructure delivery—through examining the High-Speed 2 (HS2) railway project. The article’s central contributions are twofold. First, I argue that while recent academic and political debates about judicial review’s relationship to policy making have been concerned primarily with classic questions of judicial power,^6^ the growing discourse regarding infrastructure delivery may represent a shift, accompanying the new Labour government, from a dominant political critique of judicial power towards what I term ‘litigant power’. At its core, this concerns the power which project opponents can leverage by litigating, and the delays and bureaucratic inefficiency which this induces—the critique can thus apply even to cases where courts do not find an administrative decision unlawful. I suggest this critique comprises two strands: first, engaging in the litigation process delays project implementation, even where claims ultimately fail. Second, the availability of judicial review, giving teeth to expanded legal protections of public participation, may embed overcaution among administrators and industry, thereby ‘chilling’ infrastructure delivery. The critique is increasingly expressed by think tanks like Britain Remade and UKDayOne advocating for planning reform to ‘get Britain building’ infrastructure,^7^ and raises important questions about the wisdom of trading off administrative law procedural guarantees to release the potential of (climate-friendly) infrastructure delivery in a ‘green bargain’.^8^ This is, then, an important time for public lawyers to recognise and respond to this discourse, and in effect to broaden the scholarly gaze beyond judicial power debates when examining the relationship between judicial review and policy making.

Second, having articulated this alternative form of judicial scepticism, I begin to consider the extent to which it is a legitimate concern warranting renewed judicial review reform. Using an indicative case study of judicial reviews concerning the HS2 project, I engage critically with the presence of both judicial and litigant power in the HS2 caseload. I identify little evidence of judicial overreach in the caseload to concern judicial power sceptics, but suggest there is a comparatively compelling argument that litigant power has posed problematic obstacles to delivering HS2. Nonetheless, I argue that, at least without further empirical basis, this critique may represent an oversimplistic and partial diagnosis of the problems in delivering infrastructure, and an unsatisfactory justification for the considerable restrictions on judicial review procedure it is sometimes used to support.

The article proceeds as follows. First, it articulates ‘litigant power’ as an alternative and compelling critique of the policy effects of judicial review and a changed legal culture, alongside the classic focus on judicial power. It then introduces the HS2 project within its political and public law context, before outlining the caseload of HS2-related judicial reviews. This provides context to subsequently explore the presence and impact of judicial and litigant power within the HS2 caseload. The article argues the HS2 adjudication does not evidence judicial overreach, but the litigant power critique may hold more weight. However, it suggests litigant power remains oversimplistic, particularly within a wider constitutional context wherein the national executive’s centralised decision making has arguably produced far greater inefficiencies in delivering HS2 and may pose a more significant challenge to infrastructure.

## 2. Judicial Power and Litigant Power—Alternative Critiques of a Changed Legal Culture

Much discussion of judicial review and public policy in recent years has centred on the prevalence of judicial power and the degree to which the judiciary overreaches its constitutional role.^9^ While premised on legitimate concerns, some scholars have argued that in practice the critique of judicial power in the UK has not been sustained systematically, with limited empirical evidence supporting claims that the judiciary regularly overreaches its powers—particularly when incorporating study of first-instance decision making.^10^ According to some criticism of judicial review, though, whether courts exceed their junior constitutional position, or even find in claimants’ favour, is not decisive. Rather, irrespective of individual cases’ outcomes, judicial review represents a feature of the expanded capacity for policy opponents to delay implementation, adding delays and costs, and encouraging overcaution as policy makers design schemes less amenable to litigation. This section articulates the core features of this alternative critique of judicial review’s relationship to policy making, focused more on ‘litigant power’ than judicial power, and outlines its growing political prominence. As policy commentators and government actors emphasise how public law processes may undermine efficient infrastructure delivery, we may see a subtle but important change in the dominant political engagement with judicial review in the coming years, with this alternative perspective superseding the focus on judicial power which has primarily occupied recent Conservative governments.

Litigant power’s deleterious impacts, critics argue, occur both directly, delaying project implementation while a claim goes through the litigation process, and indirectly, by encouraging (public or private) project designers to pre-emptively ‘litigation-proof’ schemes in anticipation that opponents may bring judicial review challenges against them. The 2023 Conservative government paper, *Getting Great Britain Building Again*, which preceded Lord Banner KC’s independent review on reforming judicial review of DCOs (the Banner Review),^11^ reflects this bifurcated criticism: ‘The risk of legal challenge not only compounds the risk of delays to decisions, but it has a chilling effect on actors across the whole planning and delivery system and embeds caution.’^12^ It is worth articulating each feature—the direct delay and indirect chilling effect—in turn.

### A. Litigation Delay

It is hardly controversial to note that judicial review is a temporally long process characterised by delays. This is notwithstanding the implementation of reforms, as the modern judicial review procedure has developed from 1977, that target caseload efficiency and reducing delays amid limited judicial resources.^13^ These include the permission requirement^14^ and the pre-action procedure (particularly following the Bowman Report),^15^ which exerts strict processes upon litigants and incentivises early settlement. Delay nonetheless remains a common complaint of judicial review of infrastructure projects, and more broadly: successful claimants must wait for relief, while delay heightens uncertainty and cost for decision makers and project developers.^16^ As White puts it in the infrastructure context, there is little point streamlining planning decision making if ‘endemic delays’ from the courts mean ‘judicial reviews then take six to nine months to be listed and more than a year to be finally disposed of’.^17^ Importantly, as the Banner Review notes, the time ‘lost’ is not necessarily linear, as litigation delays may intersect with other processes. Where environmental works and surveys need to be undertaken in particular seasons, even a relatively limited period of litigation delay may prevent those works from occurring until much later.^18^ Equally, a key concern is the relatively wide *variation* in delay—the time cases take to progress can be unpredictable, adding uncertainty for industry sectors reliant on predictability.^19^

### B. The ‘Chilling Effect’ of Litigation

In addition to these direct delays, critics argue judicial review induces a wider ‘chilling effect’ on infrastructure delivery. Here, citizens’ increased capacity to litigate, giving teeth to the growing procedural requirements regarding, *inter alia*, environmental impact assessment and consultation, may add internal costs, delays, uncertainty and fear to policy making and project development. In a study of US interstate construction, Brooks and Liscow have presented compelling quantitative evidence of a relationship between a growth in ‘citizen voice’ from the late-1960s and rising infrastructure costs. They define ‘citizen voice’ as an expansion in citizens’ capacity to influence government behaviour, owing to legislative changes requiring that citizens’ concerns are considered, judicial doctrine broadening their capacity to litigate and increased organisation among social movements.^20^ This increased ‘voice’ was suggested to result in government concessions, associated statistically with the adoption of expensive construction methods and mitigations such as more ‘wiggly’ or tortuous routes (intended to avoid obstacles or create scenic views).^21^ The authors questioned whether these developments accentuate the power of parties with existing resource.^22^

Crucially, in England and Wales, the capacity of citizen opponents to legally enforce procedural protections in practice is claimed to have increased over time with the relaxing of standing rules,^23^ expansion of costs protection and rise of crowdfunding.^24^ While this suggestion can itself be contested—there is much to indicate that citizens’ access to the legal expertise and funding needed to engage in both participatory planning processes and planning judicial review remains limited^25^—the prospect of litigation is conceptualised by critics as increasingly realistic and accessible, adding further unpredictability to whether projects will proceed. Administrators or developers may make concessions to claimants, uncertain whether their litigation will succeed, or adopt ‘overcautious’ internal processes before any litigation is issued,^26^ such as more rigorous impact assessment or consultation than may be legally required. These may resemble ‘shadow effects’ where, anticipating potential litigation, policy makers pre-emptively modify policy—even without any tangible challenge issued.^27^ These concerns resonate with Kagan’s classic diagnosis of American ‘adversarial legalism’.^28^ Kagan argues local citizens’ legal challenges to projects which offer public benefits but impose localised costs can create stultifying project delays and costs, primarily through encouraging designers to undertake additional ‘defensive medicine’, such as over-consulting or building mitigative structures, to minimise their legal risk amid uncertainty.^29^ Some suggest the legal uncertainty may lead investors to demand higher rates of return, so that some projects become not worth pursuing,^30^ and prevents many projects even being ‘applied for in the first place because there’s a fair chance they will be rejected’.^31^ Similarly, it is not uncommon to hear government actors express frustration that, as Dominic Cummings puts it, internal legal advice highlighting legal risk makes ‘discussion of regulatory trade-offs tortuous and wasteful; it is always easier to urge “caution” and “we’ll lose a JR” is an easy way across Whitehall to delay or block change’.^32^

Indeed, the ominous title of the Government Legal Department’s civil service guidance on administrative law, *The Judge Over Your Shoulder*,^33^ perhaps implies administrators must always remain conscious of averting legal threat. The Department for Transport seems particularly anxious of legal risk, with ‘legal concerns “front and centre”’ in policy making due to the litigious environmental sector.^34^ Policy insiders, then, appear regularly frustrated with the purported chilling effect. In the following subsection, I argue litigant power arguments are becoming increasingly prominent among policy actors, and that Labour—even if unlikely to engage in the judicial power discourse that has characterised recent Conservative engagement with judicial review—may be influenced by this alternative line of concern.

### C. Litigant Power: A New Dominant Political Critique?

Litigant power is not an entirely new political complaint, but has previously been less prominent *vis-à-vis* the judicial power discourse. Previous Conservative administrations have raised concerns associated with litigant power,^35^ with the Coalition government’s reform programme claiming litigants bring meritless cases to use ‘the delays and costs associated with judicial review to hinder actions the executive wishes to take’.^36^ Political engagement with judicial review from the political right in recent years has arguably, though, been most prominently characterised by regular criticism of judicial decisions perceived as overreaching, particularly in acutely political contexts like Brexit and immigration policy (often examining judicial reasoning in a small subset of appellate cases). Certainly, one of the Johnson government’s primary attempts to reform judicial review—establishing an Independent Review of Administrative Law in 2020 to assess the wisdom of restricting judicial review’s use as ‘politics by another means’^37^—is widely regarded as a political reaction to the Brexit-era *Miller* cases, especially *Miller II*.^38^ Here, the Supreme Court was perceived by some (including the government) to have overreached its powers in a highly fraught policy arena.^39^

Equally, the Judicial Power Project—a right-leaning think tank perceived as having considerable influence on recent Conservative governments’ approaches towards public law—has occasionally articulated concerns befitting the litigant power critique. It has raised the risk of litigants bringing unsuccessful challenges delaying legitimate policy implementation,^40^ and suggested exposure to legal risk may create a ‘chilling effect’ of risk aversion and defensive policy making.^41^ Meanwhile, Ekins and Gee, the Project’s leads, characterised the *Miller I* litigation as a legal strategy employed to delay (and perhaps frustrate) implementation of the Brexit referendum result.^42^ However, this has been far less prominent than the Project’s core focus on ‘judicial overreach’ and ‘understand[ing] and correct[ing] the undue rise in judicial power’.^43^ It is overreach, then, which under consecutive Conservative governments has arguably most animated scholars on the political right and most influenced government thinking and reform. For those sceptical of the judicial role within the constitution, the discourse of litigant power articulated here could be advanced in parallel to this more familiar judicial power discourse, as dual critiques attempting to evidence a change in legal culture—that is, to argue that the roles which law and courts play in influencing policy making and implementation have shifted.

The new Labour government, though, may less routinely critique judicial power or activism, particularly given it has expressed explicit commitment to the rule of law in its early months, perhaps in pointed contrast to its predecessors.^44^ Yet, if legalism disrupts its central policy intention to galvanise infrastructure delivery, concerns with the administrative effects of even unsuccessful litigation may increasingly influence Labour’s thinking on planning reform. Indeed, having announced it will implement some of the Banner Review’s proposed procedural reforms to judicial reviews of DCOs, Labour appears willing to, at least tentatively, trade off judicial review’s availability to deliver infrastructure.^45^ The litigant power perspective also dominates the growing calls among think tanks for Labour to restrict infrastructure litigation. A regular proposal from think tanks thus far has been to reduce the scope of judicial review on consultation grounds—consultation representing a core feature facilitating citizens’ expressions of voice. Britain Remade propose, for energy projects, that a government ‘Consultation Unit’ could ‘legally certify’ that a given project’s consultation was sufficient, to ‘radically reduce’ scope for consultative grounds of challenge.^46^ Another core theme, expressed by the Centre for Policy Studies,^47^ UKDayOne^48^ and Britain Remade’s Sam Dumitriu,^49^ has been to reform the Aarhus costs rules in environmental litigation to reduce the accessibility of challenge, with some also proposing to restrict standing. The Aarhus rules were introduced into the Civil Procedure Rules to attempt to satisfy the UK’s international Aarhus Convention obligations, that environmental justice should not be prohibitively expensive.^50^ The rules cap the government’s costs recovery from an unsuccessful claimant (and *vice versa*) in environment and planning claims. Dumitriu has suggested that Labour could disincentivise litigation by increasing the value of the capped costs which unsuccessful claimants must pay—currently £5000 for individuals and £10,000 for group litigants—or make costs protection contingent on barristers assessing a claim’s prospects of success as high.^51^ Though the government’s Banner Review found no justification for reforming the Aarhus costs rules given the risk of breaching international legal obligations,^52^ the think tanks’ recommendations represent early signs of reformist attention turning, as it often does, to ‘clamping down’ on claimants’ prospects via procedural restrictions.^53^ The proposals to restrict consultation grounds, the Aarhus rules and standing neatly reflect the core target of the litigant power critique: the legal opportunities available to environmental civil society and local groups to challenge infrastructure development.

Litigant power is, then, an increasingly prominent political argument, potentially advanced alongside or independent of judicial power concerns. The critique deserves further engagement from public law scholars, who, when considering judicial review’s relationship to policy, have arguably focused on judicial power far more than the empirical question of legalism’s administrative impacts. Given this lack of empirical focus, scholars ought to engage more closely with experiences of projects which critics present as impacted by legalism, to interrogate the legitimacy of these critiques. HS2 is one such project, often framed as plagued by legalism.^54^ Accordingly, the remainder of the article introduces HS2 and uses it as a case study to test the wisdom of both critiques of legalism—judicial and litigant power. I suggest, based on this case study, that litigant power may represent a stronger basis for concern in infrastructure decision making than judicial overreach. However, I argue the litigant power critique nonetheless appears insufficiently convincing to justify significant judicial review restrictions, particularly when placed in broader constitutional context.

## 3. Introducing HS2 in Public Law Context

This section introduces the HS2 project and its contested political progress, before contextualising it within some important public law themes.

### A. The Political Context

As initially envisaged, HS2 involved constructing an intercity high-speed railway between London and northern England, planned in phases, in a Y-shaped network. Phase 1 of the line connects London and Birmingham, and Phase 2 would continue north from Birmingham in two diverging prongs—on the western leg to Crewe and Manchester, and on the eastern leg to Leeds.^55^ Originally birthed by Lord Adonis, Labour’s Transport Secretary under Gordon Brown, the Conservative and Liberal Democrat Coalition government pushed ahead with the project following the 2010 election.

HS2 was premised on the following core justifications. First, the UK’s existing, congested railway network is approaching full capacity, particularly on the West Coast Main Line and East Coast Main Line. Limited capacity inhibits the potential to expand the number of services to meet demand, and results in trade-offs regarding which services should run.^56^ Accordingly, moving intercity rail trips to a new high-speed line would release the existing lines’ capacity to run new services, delivering more frequent and reliable commuter, regional and freight services.^57^ This capacity release should also galvanise a long-term modal shift of road and aviation passengers (and freight) onto more climate-friendly rail travel.^58^ Second, providing high-speed links between several cities in the Midlands and northern England, in Phase 2, was intended to galvanise intercity connectivity and productivity in regions outside of London.^59^ As Chancellor during the Cameron administrations, George Osborne emphasised the economic importance of creating a ‘northern powerhouse’ by improving transport networks, intending to build HS2 alongside a high-speed Northern Powerhouse Rail project connecting northern cities between Liverpool and Hull.^60^

Notwithstanding these compelling benefits, and its repeated presence within the main parties’ election manifestos during the 2010s, HS2 has throughout its lifetime faced widespread public opposition, political division and uncertainty. While some opponents have focused on its contested value for money, HS2 has faced considerable political—and at times legal—mobilisation from civil society and local communities regarding its environmental and visual impacts, especially for Phase 1 between London and Birmingham. This section passes through rural areas such as the Chilterns National Landscape and involves some tree-felling, including of ancient woodland, impacting ecosystems.^61^ Numerous concessions were thus made to allay citizen and environmental concerns, many of which were identified in the highly-detailed Environmental Statement accompanying Phase 1’s introduction.^62^ These concessions have sometimes attracted criticism as excessive, costly and overcautious ‘gold-plating’ of the project to appease expressions of citizen voice, for instance the use of expensive bat tunnels to protect rare bat species.^63^ One especially controversial mitigation was the construction of the Chiltern Tunnel for the route under the Chiltern Hills, rather than utilising a more cost-effective cutting. This concession, made during Phase 1’s authorisation in Parliament to meet stakeholder needs,^64^ brought significant expense, and is widely seen as the Conservative government appeasing strong political opposition in Conservative-voting electoral constituencies around the Chiltern Hills.^65^ As outlined below, several judicial reviews were brought by opponents and local authorities concerned with the environmental effects of features of HS2, which has intensified policy critiques of the power which opponents may leverage through political and litigious expressions of citizen voice.

The scheme’s political opposition has arguably increased the pressure on consecutive governments to curtail its scope. In late 2019, Boris Johnson’s administration commissioned the Oakervee Review to reassess the viability of delivering HS2. The Review concluded that, to optimise HS2’s benefits, it should be delivered in full.^66^ This initially sufficed for Johnson to proceed with the scheme. However, Johnson’s government later abandoned the eastern leg from Birmingham to Leeds in the 2021 Integrated Rail Plan,^67^ before Rishi Sunak also cancelled the western leg to Manchester in October 2023, meaning HS2 would stop at Birmingham.^68^ Even if ultimately delivered in full in future, HS2 is therefore likely to be regarded as a multifaceted disaster of public administration, characterised by delay, cost overruns and confusion as to the scheme’s scope amid incremental cutbacks. These cutbacks have occurred notwithstanding that, as discussed next, HS2 was approved through primary legislation.

### B. The Scheme’s Authorisation

HS2 received planning approval through Parliament, an unusual approach which offers clues as to the influence of legalism on infrastructure authorisation. Critics have long regarded approval processes as unhelpfully delaying nationally important projects, leading to cyclical rounds of reform focused on delivering infrastructure at speed. Before the 1990s, large project decision making often occurred through the ‘big planning inquiry’. This allows local actors to present their views to the Planning Inspector, and have their rights considered alongside national interests. The model had gained popularity as it facilitated citizen participation, reflecting that major planning decisions engage entire communities’ interests.^69^ Yet, during the 1980s and 1990s, this was routinely criticised as generating unacceptable costs and years-long delays.^70^ Many suggested the ‘big inquiry’ had transformed from a traditional inquiry model—balancing local rights and national interests—into an unruly, major participatory forum accepting a wide range of representations, effectively contributing to national policy formulation.^71^ Amid frustration with these costs and delays, alternative infrastructure approval processes have since been attempted, for instance the Planning Act 2008’s DCO scheme, which seeks efficiency by front-loading public participation.^72^ Another approach has been the authorisation of major rail infrastructure through hybrid Bills, a form of primary legislation, including the Channel Tunnel Rail Link Act 1996, the Crossrail Act 2008 and the HS2 hybrid Bills. In stark contrast to the big inquiry, the hybrid Bill procedure is often criticised as insufficiently participatory or deliberative.^73^ Indeed, the decision to implement HS2 via hybrid Bill was unsuccessfully challenged by judicial review in 2013, by HS2 Action Alliance and others primarily challenging its compliance with EU participation and environmental assessment requirements.^74^

To understand legalism’s potential impacts on HS2, the hybrid Bills deserve further scrutiny. The government introduced Phase 1 through the High Speed Rail (London–West Midlands) Act 2017 (the 2017 Act) and Phase 2a through the High Speed Rail (West Midlands–Crewe) Act 2021. Phase 1’s 2017 Act has been regarded as ‘one of the longest, most detailed and highly scrutinised pieces of legislation in history’.^75^ The government seemingly authorised HS2 using the parliamentary process to minimise concerns of legalism and delay associated with other approval processes. Authorisation through primary legislation circumvents the degree of local authority consultation which a route of HS2’s length would require under the Planning Act 2008 consent process^76^ and insulates the scheme’s approval from challenge on most judicial review grounds. Accordingly, we would expect little success in litigation to the scheme’s approval, potentially mitigating concerns of judicial obstruction of infrastructure. Ironically, despite these apparent attempts to mitigate the impacts of legalism, HS2’s sluggish progress has attracted complaints of cumbersome procedure, with the 50,000-page Environmental Impact Assessment for Phase 1 of HS2 commonly cited as a source of delay.^77^ There is a sense of *déjà vu* in criticisms that planning processes complicate and delay infrastructure delivery. Even where efficiency-oriented approaches are adopted—whether hybrid Bills or DCOs—complaints often arise that pre-decision participation and post-decision litigation induce delays.

This is an apt time to assess the 2017 Act’s effectiveness in mitigating bureaucratic delay and disruption to HS2 from judicial review. This is because commentators are beginning to encourage the Labour government to authorise new rail projects through hybrid Bills, to render them ‘essentially immune to judicial review’.^78^ However, even if HS2’s legislative approval has rendered its *authorisation* relatively immune to challenge, it is unclear how far the hybrid Bill has minimised challenges to features of HS2’s administrative *implementation*. While the legislation provided high-level authorisation of HS2’s construction, many features of HS2’s implementation could not have been planned for in detail during its parliamentary approval, such as localised works in particular locations. Accordingly, the 2017 Act created a bespoke legislative scheme for HS2’s administrative implementation, providing executive bodies with powers under the Act. One such example, discussed below, is the process established in Schedule 17 of the Act, whereby HS2 Limited, the executive body delivering HS2, needs to secure planning consent from particular local authorities to carry out works in their area. That HS2 has needed to obtain over 8000 individual consents after its top-level legislative authorisation has itself received think tanks’ criticism, urging any new legislation authorising infrastructure to ‘minimise, if not remove, the need for secondary approvals’.^79^ There may therefore be instances where the judiciary, tasked with interpreting the legality of executive action implementing the complex legislative scheme, exerts influence on HS2’s implementation. To explore this, the next section introduces the caseload of HS2-related judicial reviews, underpinning the subsequent analysis of judicial power and litigant power.

## 4. Introducing the HS2 Caseload

To explore judicial review’s role in the delivery of HS2, and engage with the increasing critiques of litigant power, this study has located all publicly accessible judicial reviews related to features of HS2’s authorisation and implementation. Though HS2 has been litigated in several contexts, including statutory appeals and protest cases, this study focuses on judicial review, given its prevalence in policy critiques of infrastructure delivery. A keyword search was conducted across the legal databases BAILII, Westlaw and LexisNexis, confined to litigation in the Supreme Court, Court of Appeal (Civil Division) and High Court.^80^ This search included many non-judicial review cases, which were reviewed and removed. The search was supplemented by internet searches. Fourteen judicial reviews were ultimately located—though others, especially if resolved at early stages, could have been unreported and unrepresented. Equally, pre-action protocol letters may have been sent and resolved or abandoned before reaching the courts, which this cannot capture.

Of the 14 claims, three can be characterised as successful and 11 unsuccessful. Success and failure occurred at different stages of the litigation process. Five were refused permission (whether on the papers or when orally renewed) and proceeded no further,^81^ while one claim unsuccessfully sought an interim injunction and was seemingly discontinued afterwards.^82^ Five lost at a substantive hearing,^83^ including the first *HS2 Action Alliance* claim (*HS2AA 1*) in 2013, which reached the UK Supreme Court the following year and has become a significant constitutional law decision.^84^ This claim challenged the decision to authorise HS2 via hybrid Bill, largely for breaching EU environmental law. One somewhat marginal ground, related to HS2’s consultation around blight provisions, was successful before the Administrative Court and was not appealed by the defendants. However, the claimants nevertheless appealed the Administrative Court decision, and lost in the appellate courts on all other grounds, so this appears unsuccessful. Only one case succeeded at substantive hearing,^85^ while one claimant settled favourably out of court before the permission stage.^86^ Finally, a 2014 claim, *Thornton*, challenged a decision notice by the Transport Secretary which overrode the Information Commissioner’s decision to release a 2011 Project Assessment Report on HS2. The claim was granted permission but stayed pending the UK Supreme Court’s *Evans* judgment regarding the ministerial veto power.^87^ The outcome of *Evans* was favourable to the claimants in *Thornton*, meaning the government conceded the claim, with the project report released.^88^ Across the caseload, the Secretary of State for Transport was a (co-)defendant nine times and an interested party once; and HS2 Limited was a (co-)defendant four times and an interested party six times. The Secretary of State for Housing, Communities and Local Government was the next most frequent defendant, appearing twice.

The dates of the litigation and the characteristics of the claimants themselves are notable as regards HS2’s experiences with adversarial legalism. A few claims arose during the early stages of HS2’s passage through Parliament—including the above *Thornton* and *HS2AA 1* claims, and a second claim by HS2 Action Alliance and others in 2014 challenging the safeguarding directions for properties along the proposed route (*HS2AA 2*).^89^ No challenges were located, though, between 2016 and 2019, before a plethora of claims from 2019 onwards. This may indicate that, at least following the Supreme Court’s assent to the hybrid Bill procedure’s design in *HS2AA 1*, HS2’s parliamentary approval proved largely effective in minimising litigation contesting its *authorisation*. Then, from 2019, several discrete features of HS2’s executive *implementation* were challenged, across contexts including procurement, tree licence decisions and local authority approvals. While almost certainly adding cost and delay per the litigant power critique, challenges to features of implementation are perhaps inevitable in an enormous, complex scheme engaging numerous administrative decision-making contexts.

However, legal contestation over HS2’s authorisation was reignited in 2020 after Boris Johnson commissioned the aforementioned Oakervee Review to consider whether to continue the project. Some opponents saw Johnson’s decision to continue with HS2 following the Review as an opportunity to relitigate the merits of authorising HS2. Chris Packham challenged the Review’s process and Johnson’s subsequent decision—effectively challenging the scheme’s approval itself—but was refused permission by the High Court^90^ and the Court of Appeal.^91^ Packham argued that the Review’s process did not comply with its Terms of Reference because a key panel member had departed, and that this breached the public’s legitimate expectation of the Review’s scope. The courts noted the Review was not a statutory creation, had a limited role and scope, and the process and Johnson’s subsequent decision were macro-political, providing room only for a low-intensity rationality challenge. Packham also argued the Review failed to account for local environmental and climate change concerns. The courts noted the Review’s limited Terms of Reference did not require detailed assessment of environmental impact, suggesting this argument was illegitimately reopening the legislative debate preceding the 2017 Act. Later in 2020, following the Review, the government issued a Notice to Proceed with HS2. Joe Rukin, manager of the prominent StopHS2 campaign, challenged the Notice to Proceed, and was refused permission on the papers. Rukin’s grounds were regarded as simply ‘points on which the claimant disagrees with’ HS2, some grounds re-ran ‘matters considered and rejected’ in *Packham* and one ground alleged the officials presenting HS2’s business case contravened the Fraud Act 2006.^92^ Some campaigners, then, litigated to undermine HS2’s legislative authorisation, a stark attempt at adversarial legalism which the courts clearly recognised, with neither claim granted permission. Equally, though, Johnson’s uncertainty whether to proceed led him to commission the Oakervee Review, indicating government indecision also delayed and disrupted HS2. The Review’s recommendation to deliver HS2 in full as already planned makes this delay appear especially wasteful, particularly as it also provided windows of opportunity for opponents to litigate.

The claimants themselves tended to fall into one of three categories. First, two companies—Patentes Talgo in 2021 and Siemens in 2023—challenged procurement processes for the manufacture and supply of HS2 trains, having unsuccessfully sought tender. These relatively recent challenges addressed discrete executive decisions implementing HS2—rolling stock procurement processes—rather than project authorisation. Both claims were launched alongside procurement challenges in the Technology and Construction Court under Part 7 of the Utilities Contracts Regulation 2016 (UCR). Talgo settled its claim out of court.^93^ Siemens’s judicial review grounds mirrored the grounds in its (unsuccessful) Part 7 challenge—including alleging a breach of Siemens’s legitimate expectations—but on the basis that HS2 breached public law duties rather than duties under the UCR.^94^ The court, refusing Siemens permission, held judicial review was an inappropriate remedy—the issue did not contain any public law element, and the Part 7 proceedings should have been exhausted first as judicial review is a remedy of last resort.^95^

Second, and more regularly, a range of campaigners litigated HS2,^96^ usually on environmental grounds, with many funding their claims using crowdfunding. For instance, in 2021, ecologist Mark Keir was refused permission in arguing Natural England had erred in granting HS2 licences to fell trees in an area where one tree to be felled had the potential to support a barbastelle bat breeding site.^97^ Under the Conservation of Habitats and Species Regulations 2017,^98^ Natural England must be satisfied it has no reasonable scientific doubt that the licensed actions would not be detrimental to maintaining the bat population at a favourable conservation status. Presenting text from Natural England’s assessment documents, Keir argued it could not have had no reasonable doubt of this. The court noted this evidence was ‘wrenched out of context’ in a ‘highly selective filleting of the material and an excessively legalistic or forensic approach’.^99^ Other grounds alleging Natural England departed from its policies and acted irrationally also involved unduly legalistic failures ‘to read both that material and the decision as a whole’.^100^ Also in 2021, a Buckinghamshire-based community group, Misbourne Environmental Protection Ltd, was refused permission to challenge HS2’s assessment of water quality effects in the River Misbourne arising from groundwater construction for the aforementioned Chiltern Tunnel (which, following citizen pressure, was being constructed to carry trains beneath the Chiltern Hills).^101^ Relying on the Water Framework Directive 2000/60/EC, the group argued the tunnelling risked contaminating water, but the court noted the Directive is concerned with a deterioration in the water body’s longer-term ‘status’, whereas any risks here appeared temporary and thus would not impact the water body’s ‘status’.^102^ Some claims, such as Rukin’s 2020 challenge to the issuing of the Notice to Proceed with HS2, lacked arguable legal basis and were evidently little more than instrumental attempts to disrupt the project. Not all project opponents’ challenges, though, were hopeless claims. Dr Paul Thornton, who has published for StopHS2 and petitioned against the 2017 hybrid Bill,^103^ was a claimant in *Thornton*, where a Project Assessment Report was disclosed following an out-of-court settlement, litigating to optimise freedom of information and executive accountability.^104^ Meanwhile, HS2 Action Alliance’s first claim succeeded on one ground in the Administrative Court^105^ and, while otherwise unsuccessful, was sufficiently arguable to reach the Supreme Court and enter the constitutional law canon.

Interestingly, some judicial review claimants, including Mark Keir and Larch Maxey, also appeared as named defendants in injunction proceedings concerning their anti-HS2 protests.^106^ Eliding the classic distinction between proactive and reactive uses of litigation within social movements,^107^ they invested hope in law’s justice while being subject to its enforcement. In launching judicial review proceedings in 2021, Maxey sought an interim injunction to cease operations extracting him from a tunnel which he was occupying in protest, on land temporarily in HS2 Limited’s possession.^108^ This injunction was refused, Maxey was extracted from the tunnel and the judicial review was discontinued. Maxey, though, later appeared in contempt of court proceedings regarding breaches of the court order made in the interim judgment.^109^

Third, local authorities brought five challenges as (co-)claimants,^110^ most notably London Borough of Hillingdon Council and Buckinghamshire Council. Both were heavily involved in political opposition to HS2, organising 51M, a coalition of 18 local authorities opposed to the scheme, 15 of whom were co-claimants in *HS2AA 1*. Three claims by local authorities concerned the role of local planning authorities (LPAs) in approving localised construction works under the 2017 Act. To minimise project delays, the Act streamlined the process by which LPAs approve works. Section 20 deems that HS2 Limited—the executive body carrying out works—is granted planning permission for works relevant to constructing HS2, subject to conditions in Schedule 17. Under these conditions, HS2 Limited must seek approval for developments within an LPA’s boundaries (including building work, earthworks, fences and noise screens) from that LPA. The LPA has an eight-week period to determine requests, and its powers to refuse approval are highly circumscribed. On three occasions (Hillingdon twice and Buckinghamshire once), LPAs refused approval for works, which HS2 successfully appealed before the Secretary of State,^111^ leading the LPA to challenge that appeal decision by judicial review.

In *Hillingdon 1*, HS2 Limited had sought Hillingdon’s approval for earthworks and fencing supporting a new wetland habitat. Hillingdon asked HS2 to provide additional information on ecological and archaeological impact, which HS2 did not submit, and Hillingdon recommended refusal. HS2 successfully appealed that decision, and Hillingdon brought a judicial review challenging the appeal decision. Hillingdon’s judicial review primarily concerned statutory construction: did Schedule 17 of the 2017 Act require an LPA to approve HS2’s requests even if the LPA lacked the evidence necessary to perform an assessment of impact? The Administrative Court dismissed the claim, noting the Act gave LPAs an ‘unusually restrictive’ decision-making role and HS2 had provided sufficient information for Hillingdon to perform that circumscribed role.^112^ The Court of Appeal, though, allowed Hillingdon’s appeal, firmly articulating how Parliament gave LPAs ‘democratic responsibility and accountability’ to assess works’ impacts on specified planning interests.^113^ HS2 therefore could not decline to provide the evidence alongside its application which the LPA required to make a lawful approval decision under its statutory duty.^114^ The Court also recommended that in future, where HS2 does not provide sufficient information for a rational decision maker to exercise its statutory functions, the LPA should—rather than refuse the approval request—decline to process it until adequate evidence is provided, thereby preventing the eight-week determination period from commencing.^115^ Beyond the extreme circumstances of *Hillingdon 1*, where HS2 had provided no requested information, it was uncertain how far this recommendation would apply, and it appeared to empower LPAs in future to more readily refuse or decline to approve works. This uncertainty was addressed in further litigation, first in *Hillingdon 2* in 2021. Hillingdon had refused HS2 approval to construct lorry routes related to five construction sites, claiming HS2 had not submitted information supporting its traffic management arrangements during peak periods and had declined to accept two conditions to prevent road traffic effects. HS2 successfully appealed this before the Planning Inspector, and Hillingdon challenged that appeal decision by judicial review. The Administrative Court dismissed the claim,^116^ and the Court of Appeal refused permission to appeal.^117^ In the Administrative Court, Ouseley J discussed the Court of Appeal’s recommendation from *Hillingdon 1* to decline to approve requests, regarding it as obiter and expressing reservations that it could not be applied beyond that extreme case without unintended collateral litigation and delays.^118^ The Court of Appeal in *Hillingdon 2* acknowledged Ouseley J’s concerns, but did not resolve the tension between his comments and those in *Hillingdon 1* as it was not decisive.^119^ This resolution later arose in *Buckinghamshire* in 2022.^120^ Buckinghamshire Council, seemingly encouraged by the recommendation in *Hillingdon 1*, had declined to determine seven lorry route approval requests altogether, stating the information HS2 had provided was insufficient. After the Planning Inspector allowed HS2 Limited’s appeal, Buckinghamshire brought an unsuccessful judicial review of the Inspector’s decision. It contended that, having declined to determine the requests, the eight-week period for determination had never commenced, meaning the Planning Inspector lacked jurisdiction to hear an appeal. This argument found no basis in the statutory scheme or *Hillingdon 1*, and other arguments that the Inspector erred in his assessment, including erring in the weight he placed on certain documents, were dismissed as challenges to his planning judgment, which, short of an error of law or irrationality, the court should not interfere with. In *Hillingdon 2* and *Buckinghamshire*, the courts expressed unease that the LPAs relied on the recommendation from *Hillingdon 1*, a case where HS2 Limited had provided none of the information requested, to argue in entirely different circumstances (where HS2 had provided information) that the Planning Inspectors’ decisions were made unlawfully with insufficient evidence. The question of whether the Inspectors had sufficient information to make a decision was a matter of planning judgment. Though these cases addressed complex questions, then, there was sometimes even here arguably an instrumental flavour.

What emerges across these profiles is a series of concentrated, often locally based interests, many concerned with HS2’s environmental impact. Kagan regards such interests as those most prone to adversarial legalism.^121^ Having traced the HS2-related caseload, the following section further explores the courts’ decision making, arguing that—contrary to concerns of judicial power—this adjudication presents little evidence of overreach.

## 5. Judicial Power in the HS2 Caseload

The article now scrutinises the courts’ work in the HS2 caseload, to explore whether a critique can be sustained on the basis of classic concerns of judicial overreach. Aligning with much of the empirical evidence on judicial power in recent years,^122^ the story of judicial intervention in HS2 was largely unremarkable. The courts frequently filtered out interests at early stages, and only very rarely did grounds of challenge meet doctrinal standards.

As discussed above, the courts at numerous points heard claims effectively attempting to litigate the merits of a project already assessed in detail and authorised by Parliament, or to scrutinise the (expert) judgment of administrators implementing the scheme. Several judges emphasised the ‘considerable weight’ attached to HS2 progressing ‘without substantial interruption’, given ‘Parliament has decided that it is in the public interest’.^123^ In *Packham*, the High Court and Court of Appeal, refusing permission to challenge the decision to continue with HS2 following the Oakervee Review in 2020, appeared conscious that the claim resembled a backdoor challenge to ‘re-run’ Parliament’s approval of HS2. When arguing that the Review erred in its consideration of local environmental impacts, Packham’s counsel accepted there was nothing in this ground which Parliament had not already considered.^124^ The High Court emphasised, in notably robust terms, the ‘strong public interest in ensuring that, in a democracy, activities sanctioned by Parliament are not stopped by individuals merely because they do not personally agree with them’.^125^ Indeed, in *Hillingdon 1*, the only claim which was successful in court, the Court of Appeal held HS2 Limited prevented Hillingdon from performing its duty under the 2017 Act by providing no information alongside its application—HS2’s conduct was contrary to the thrust of Parliament’s intent. Here, the court constructed the statutory scheme to give effect to, rather than frustrate, Parliament’s intention,^126^ that local authorities be involved in managing localised impacts of project implementation which Parliament had not planned for in detail by 2017. By contrast, the courts were subsequently reluctant, in *Hillingdon 2* and *Buckinghamshire*, to facilitate LPAs relying on the *Hillingdon 1* judgment to act and litigate in a manner arguably obstructive of the Act’s purpose and to question Planning Inspectors’ expert planning judgment. A possible explanation for claimants’ limited success, and the courts’ minimal intervention, is therefore a judicial fidelity to the legislative scheme.

A recurring theme concerned judicial review’s doctrinal boundaries where claimants appeared to contest decisions’ merits. It is hardly uncommon to see merits challenges shoehorned into planning judicial reviews—third parties have limited appeal rights in planning, so have little option beyond judicial review.^127^ Several claimants relied on bare rationality challenges without approaching the high doctrinal *Wednesbury* threshold,^128^ including in areas where the doctrine encourages a particularly light-touch approach, such as where decision makers exercise planning judgment^129^ or expert scientific judgment,^130^ or make macro-political decisions.^131^ Multiple claims contested the merits of the expert or scientific judgment of executive officers implementing the scheme, and the courts were reluctant to engage substantively with the considerable factual evidence and witness statements at times presented to support those contentions. In *Keir*, Holgate J, refusing permission to challenge Natural England’s 2021 grant of tree-felling licences to HS2, regarded the expert ecological evidence which Keir presented as ‘largely directed at challenging the merits of the judgments reached by [Natural England] and advancing alternative expert opinions’.^132^ In *Misbourne EP*, where the claimant unsuccessfully sought permission in a claim arguing the risk of contamination affected the River Misbourne’s ‘status’, its submissions were at points regarded as contesting the environmental expertise and judgment of HS2 and Environment Agency staff as to the extent and duration of the contamination risk.^133^ In *Granger-Taylor*, at substantive hearing in 2020, the claimant unsuccessfully challenged the design of the approaches to London Euston station under Article 8 and Article 1 Protocol 1 (A1P1, the right to property) of the European Convention on Human Rights (ECHR), arguing the design risked catastrophic collapse to a retaining wall that would seriously damage her property. This raised much complex engineering evidence contesting the design’s safety, causing Jay J to observe the ‘obvious dangers of the court overreaching itself by delving into issues which are way beyond its competence’.^134^ The claimant’s request that the court compel HS2 Limited to re-embrace an alternative design it had previously rejected also exceeded the court’s supervisory jurisdiction.^135^ Meanwhile, in the 2021 interim relief decision in *Maxey*, Maxey contested the factual evidence of HS2’s extraction staff regarding the safety of extracting him from the tunnel he occupied in protest. Maxey relied heavily on a tunnel removal specialist’s witness statement. The court declined to reject HS2’s factual evidence, from those present on site, based on the ‘concerns and beliefs’ of someone who had ‘not been to the site’.^136^ In short, several challenges straightforwardly failed to meet the doctrinal standards by which the courts are limited, and the courts appeared conscious of merits challenges and contestations of expert judgment.

It is also noteworthy, given the extent HS2’s statutory scheme engages property rights, that the courts’ engagement with property rights arguments has been relatively light-touch. Echoing classic left-wing judicial scepticism that the courts’ protection of private property can encourage decisions contradicting the thrust of legislation furthering the public good,^137^ some fear that A1P1 ECHR jurisprudence may disrupt social legislation, most clearly through challenges to compulsory purchase schemes.^138^ Yet the courts were rarely invited to address A1P1 in judicial reviews concerning HS2. In Ouseley J’s 844-paragraph Administrative Court judgment in *HS2AA 1*, A1P1 was only raised by the claimants to supplement one ground which alleged a failure to re-consult on route amendments that would adversely impact some properties, and Ouseley J quickly dismissed its significance.^139^ Nor did A1P1 arise in *HS2AA 2* in 2014, when HS2 Action Alliance and others unsuccessfully challenged the directions for safeguarding the route.^140^ As with *HS2AA 1*, this claim primarily drew upon the EU’s Strategic Environmental Assessment Directive. The court engaged with A1P1 in *Granger-Taylor*, regarding the Euston approaches design, but afforded the state a ‘fairly broad’ margin of appreciation in undertaking a proportionality analysis, concluding the design would not impose a ‘disproportionate or excessive burden’ on Granger-Taylor, guided by relevant A1P1 jurisprudence.^141^

In sum, the courts have decided these claims in an orthodox and uncontroversial manner. Claims rarely approached the relevant thresholds for the grounds of review, while some risked contesting decisions’ merits, or engaged decision-making contexts where the case law encourages low-intensity review. Amid popular concern that judicial review may obstruct infrastructure delivery, it is important to recognise that we do not see egregious overreach. Meanwhile, in the one successful claim, the court realised Parliament’s intent through statutory construction. Much of the courts’ work was managerial, occurring at early stages. Citizens’ complaints could be aired in an official channel and processed decisively—arguably an underappreciated facet of judicial review—but the courts adhered to a ‘strong filtering’ process, managing stakeholders’ interests from the system fairly and efficiently.^142^ These experiences may support the hypothesis that the hybrid Bill procedure mitigates the risk of successful litigation: even in claims challenging administrative implementation rather than authorisation, the public interest in avoiding disruption to a scheme approved by Parliament appeared to weigh strongly on the courts, regularly adopting a minimalist approach in a ‘sensitive policy environment’.^143^ This potentially strengthens the case for new rail infrastructure to be implemented through hybrid Bills, in minimising successful challenge.^144^ While a critique of judicial power is difficult to sustain in HS2, though, litigation outcomes are only part of the story of judicial review’s effects on infrastructure. The next section turns to the alternative litigant power critique.

## 6. Litigant Power in the HS2 Caseload

The discussion until now has detailed an orthodox and minimalist pattern of adjudication, providing little evidence to support classic critiques of overreach: claims very rarely met the thresholds for grounds of review, and the hybrid Bill drastically minimised legal contestation over project authorisation. This might indicate judicial review represents little threat to infrastructure development, at least for the statutorily approved HS2. Yet the alternative litigant power critique outlined in section 2, highlighting delays and internal chills associated with the litigation process, can in some respects be made irrespective of claims’ outcomes. Indeed, governments and think tanks advancing such complaints have commonly foregrounded delays from entirely meritless and unsuccessful litigation. Discussing HS2, UKDayOne note that while ‘courts have generally found in favour of the government, these legal challenges have contributed to delays, increased costs and [HS2’s] eventual scaling-back’.^145^ Here, I address each strand of litigant power in turn: litigation delay and then the chilling effect. I argue litigant power represents a stronger critique of legalism’s impact on HS2 than judicial power, but remains oversimplistic and currently insufficiently convincing to justify the significant reforms sometimes proposed.

### A. Litigation Delay

This subsection discusses the prevalence and impact of litigation-induced delay in the HS2 caseload, and wider questions of delay in planning claims.

#### (i) Delay and expedition in HS2

It might provide critics of litigant power some solace that judges were routinely alive to risks of administrative delay to HS2, and applied their discretion in managing the caseload to mitigate this while protecting the rule of law. Expedited hearings for claims were regularly ordered,^146^ including ordering an unusual, expedited rolled-up permission to appeal hearing in the Court of Appeal in *Packham*.^147^ This emphasis on expedition is forcefully demonstrated by the various interim injunctions which claimants sought, attempting to prevent HS2’s clearance works. In *Keir*, alongside seeking permission, Keir requested an injunction to prevent tree-felling pending a full judicial review hearing. The permission and injunction hearing took place on 23 April 2021, and the barbastelle bat maternity season was presumed to occur between May and October. Holgate J was clear that had the judicial review been arguable, which it was not, the balance of convenience would firmly favour discharging the injunction. Considerable weight was attached to the estimated costs of £60.7–88.8 million from delaying tree-felling until October, after the maternity season, which would also have prevented earthworks commencing until spring 2022.^148^ Litigation delay would thus have been non-linear, compounded by works needing to take place seasonally.^149^ The costs of similar delays also influenced the balance of convenience in refusing an interim injunction to prevent clearance works in six ancient woodlands in *Packham*—alongside the democratic risk of disrupting works ‘long ago authorised by Parliament’.^150^ The Administrative Court also considered Packham was not prompt in issuing his claim (although the Court of Appeal disagreed), noting delayed issuing risked disrupting the completion of clearance works by 5–6 months.^151^ Though the defendant had not contested Packham’s promptness, the court felt it necessary to address this,^152^ enforcing procedural rigour.^153^ These accounts indicate the litigation process is capable of causing costly delays, but also reveal a judicial emphasis, particularly within early-stage adjudication, on expedition and minimising such risks.

Yet, even if the courts prioritise efficient caseload management, some may question how far delay can be mitigated without reducing that caseload, whether by restricting standing, time limits or costs protection, or by ousting justiciability for infrastructure challenges. The use of hybrid Bills arguably reflects this intention, excluding most grounds of challenge to the scheme’s authorisation. It is notable, then, that although most have been unsuccessful, HS2 has nevertheless faced numerous claims and has not been immune to litigation-induced delays. Critics often emphasise the delays from a litany of meritless claims,^154^ and could highlight several claims by environmental opponents which were refused permission but will have nonetheless added some degree of disruption to HS2’s implementation—including *Keir*, *Packham*, *Misbourne EP* and *Rukin*. There is, though, a challenge here for critics of litigant power. Such meritless claims are resolved relatively expeditiously. The claims which likely take longer to conclude are those reaching substantive hearings—precisely because, even if ultimately unsuccessful, they had arguable grounds with a realistic prospect of success. The regular criticism of an increase in meritless claims perhaps represents a more palatable justification for arguments to reform judicial review procedure—positioning the system as being misused for frivolous campaigning—but is rarely evidenced empirically, and permission stage statistics do not systematically support arguments of increasingly meritless litigation.^155^ A more transparent, but more politically difficult, argument would perhaps acknowledge that courts filter out meritless claims at early stages, but argue judicial review’s ordinary operation nonetheless delays and chills infrastructure, meaning restrictions are needed in a ‘green bargain’.^156^ Such arguments may be ill-advised, given their implications for the rule of law. However, judicial reviews, whether arguable or not, will to some extent add time and uncertainty for infrastructure delivery. It is, then, reasonable to consider the feasibility of institutional reforms which do comply with the rule of law but reduce uncertainty and encourage timeliness.

#### (ii) Systemic delays in infrastructure claims

In this regard, we enter familiar territory from a decade ago, when infrastructure delays again reached the political agenda. Within a broader programme of judicial review reform, the Coalition government in late 2013 highlighted the risk of infrastructure delays from planning claims, and proposed creating a Specialist Planning Chamber within the Upper Tribunal hearing planning judicial reviews and statutory reviews, targeting efficient resolution and greater certainty for industry and local residents.^157^ This was consulted on notwithstanding that the Administrative Court, alive to the importance of resolving planning claims speedily, had established the ‘planning fast-track’ in July 2013, which flagged judicial review and statutory challenges to major projects to be expedited or heard by a specialist judge.^158^ Regardless, the government’s consultation led to the Planning Court being established, operating as a separate list within the Administrative Court with a stricter timetable, and ensuring judges with planning expertise hear cases, to increase efficiency.^159^ The Planning Liaison Judge in charge of the Planning Court can also designate cases as ‘significant’; such cases are further expedited with strikingly demanding target timescales,^160^ and early data on the Court’s efficiency was very positive.^161^ The degree to which this has encouraged more efficient resolution of infrastructure challenges is important, in a debate at present characterised more by assertion than by empirical evidence. Table 1 is taken from the Ministry of Justice’s quarterly judicial review statistics up to 2024 (Quarter 2).^162^ It presents the time (in days) from lodging a claim to important milestones in litigation, across the caseloads for town and country planning; town and country planning (significant); and transport (non-RTA). As an indicative comparison, it presents the timescales between 2000 and 2024, and between 2013 (Quarter 3) and 2024. Quarter 3 of 2013 was the first quarter when a case designated as ‘significant’ was lodged. The table also isolates these ‘significant’ cases from 2013 to 2024.

**Table 1.** Days from lodging to case milestones (judicial review civil justice statistics, Ministry of Justice)

	2000–2024 (Q2) (town and country planning; town and country planning (significant); transport (non-RTA))	2013 (Q3)–2024 (Q2) (town and country planning; town and country planning (significant); transport (non-RTA))	2013–2024 (Q2) (town and country planning (significant))
Lodging to permission	83.6	71.8	80.4
Lodging to renewal	163.1	133.7	125.8
Lodging to final hearing	262.5	229.5	216.2
Lodging to case closed	213.4	178.4	200.5

Albeit an imperfect comparison, these statistics do indicate somewhat more efficient case management since the Planning Court’s establishment, including among ‘significant’ cases. Equally, the Banner Review provided evidence, for DCO challenges under the Planning Act 2008, that the High Court was on average close to meeting the target timescales, but Lord Banner noted there were at times some considerable deviations from those averages which would heighten uncertainty.^163^ Progress with expedited listings in the Planning Court may, though, be undermined if litigation reaches the Court of Appeal,^164^ which faces chronic delays listing any subsequent appeal.^165^ Notably, the Banner Review has recommended introducing target timescales in the Court of Appeal for DCO claims.^166^ The tentative progress in the Planning Court indicates that credible solutions may exist that are capable of alleviating some of judicial review’s delays while complying with the rule of law, and there is scope for further consideration of such options. For instance, during the 2013 reform cycle, Fordham and others provided several rational options to streamline judicial review while protecting the rule of law,^167^ representing viable approaches to improve system efficiency without undermining access to justice. Some would perhaps surprise reformists—such as defendants and interested parties more regularly conceding rather than resisting permission, something the Banner Review rightly echoes.^168^ If the current Labour government does look to further modify judicial review within its planning reforms, beyond the changes it is implementing following the Banner Review, it could do much worse than revisiting Fordham and others’ proposals. Of the recommendations from the Banner Review which the government is introducing, there are several sensible procedural suggestions, such as introducing automatic pre-permission Case Management Conferences in judicial review claims challenging DCOs, and removing the paper permission stage in these claims.^169^ These proposals may offer developers some improved certainty and predictability as to timescales. Yet they tinker at the edges of judicial review reform, and it is difficult to envisage them quelling the yearning of politicians and think tanks to release infrastructure delivery.^170^ Moreover, the similarity of the 2013 reform period with current debates suggests that we have been here before. Concern around delay from planning judicial review appears cyclical, as it does for approval processes such as ‘big inquiries’.^171^ What goes unaddressed are more fundamental questions regarding adequate funding of the court structure to efficiently process backlogs. There is little point providing target timescales at the Court of Appeal, for instance, if wider resource backlogs and bottlenecks undermine its ability to meet those timescales. Furthermore, the cyclical focus on litigation delay perhaps represents something of a distraction which does not get to the heart of inefficiencies within infrastructure delivery. Indeed, as discussed later, the centralisation of executive decision making may be an under-theorised, but more problematic, public law cause of infrastructure delays.

### B. The ‘Chilling Effect’ of Judicial Review

#### (i) HS2 and legal risk

The direct delay resulting from litigation is only one feature of the complaint around infrastructure judicial review. Litigation is also said to embed a cautious ‘chilling effect on actors across the whole planning and delivery system’.^172^ This argument is partly concerned with how legalism impacts project delivery in unseen ways, in the shadow of (potential) litigation and legal risk. Indeed, one of its most compelling strands is incredibly difficult to quantify: the extent to which legal uncertainty has made it ‘near impossible’ to plan investment decisions, freezing some projects from proceeding whatsoever.^173^ Because these growing concerns are difficult to quantify, however, the chilling effect is empirically somewhat nebulous, making it an uncomfortable basis on which to ground judicial review reform restricting litigants’ prospects. It is also difficult to persuasively confirm or falsify through the sort of case law analysis pursued here, calling for more dedicated empirical inquiry.

Nevertheless, in some senses, the case can be made quite straightforwardly and self-evidently that features characteristic of litigant power will have directly added costs and time to HS2’s delivery. Take the decision to introduce HS2 via primary legislation. Both the Phase 1 (London to Birmingham) and Phase 2a (Birmingham to Crewe) hybrid Bills took well over three years to pass from publication in Parliament to Royal Assent, with both highly scrutinised in Parliament. A House of Commons HS2 Select Committee was established for the Phase 1 Bill, to alleviate citizen stakeholders’ localised concerns, which heard evidence from 1600 petitioners and encouraged HS2 Limited to agree deals with petitioners in the corridors outside the committee room to neutralise their concerns.^174^ The hybrid Bill, it has been suspected, was in part adopted to mitigate requirements of consultation along the route and the risk of litigation, arguably representing an internal mitigative choice made in the shadow of legal risk. Accompanying the hybrid Bill was a 50,000-page environmental assessment. The assessment was required under EU law,^175^ a requirement given teeth by the prospect of domestic judicial review. Meanwhile, though the Administrative Court gave short shrift to A1P1 ECHR considerations in *HS2AA 1* and adopted a wide margin of appreciation when considering A1P1 compatibility in *Granger-Taylor*, we might expect that the *prospect* of A1P1 litigation (alongside political demands) will have weighed on the designers of, for instance, the discretionary compensation scheme for owners of compulsorily purchased property. Notably, this scheme went beyond the minimum requirements of compulsory purchase law,^176^ potentially encouraging suggestions that government made expensive concessions in anticipation of citizen voice and litigant power. For both the environmental assessment and design of the compensation scheme, the government very likely adopted more stringent processes (at least in part) to alleviate legal risk, perhaps exhibiting the policy impacts of a changed, Europeanised legal culture which has broadened the legal stock for rights protection and citizen participation.

We may also see citizens reinforce political action through litigation. The Conservative government’s perceived need to allay citizen concerns in traditionally Conservative-voting constituencies in southern England surely increased costs, especially when constructing the Chiltern Tunnel as a visual shield in Buckinghamshire,^177^ rather than using a cutting. While influenced by a leverage of citizen voice, this has not resulted from litigation and is not itself relevant to a critique of judicial review. However, the Environment Agency’s consent to the precise tunnelling arrangements in the Chilterns was later itself unsuccessfully challenged by judicial review in 2021, by the Misbourne Environmental Protection community group,^178^ which certainly adds to a perception that localised expressions of voice can be reinforced by the increasingly accessible prospect of litigation.^179^

While some features are by their nature empirically difficult to evidence (or rebut), the chilling effect’s concerns are weighty. Administrative law procedural requirements—and their prospect of litigious enforcement—will have certainly added burdens to HS2’s design and delivery. As discussed next, though, there are risks in too narrowly diagnosing problems with delivery to government overcautiously ‘proofing’ decision making from successful litigation.

#### (ii) The analytical limits of the chilling effect

In this regard, I tentatively argue, based on HS2, that the complaints of a chilling effect may be oversimplistic, with its complainants overstating the benefits and underestimating the costs of loosening infrastructure delivery from legal risk. For a complaint that emphasises looking beyond individual judicial reviews to the wider administrative impacts of adversarial legalism, it ironically risks overlooking the broader political contexts within which litigation takes place. At least four points can be made here.

First, the chilling effect framing obscures the possible instrumental role of participation in delivering good outcomes,^180^ that thorough participatory practices and the prospect of their judicial enforcement improve decision-making quality and ensure infrastructure is delivered with appropriate regard for affected publics. This is not to deny there may be legitimate concerns regarding spiralling project costs and delays. We might question the relative value to good administration of HS2’s 50,000-page Phase 1 Environmental Statement, or whether constructing tunnels at exorbitant cost to remove the railway from view or protect rare bat species was proportionate to alleviate social costs.^181^ Meanwhile, the judicial discussion in *Keir* details a burdensome degree of mitigation, undertaken on the worst-case assumption that one maternity roost of barbastelle bats was present—it was unclear whether this roost in fact existed.^182^ Yet extensive consultation and assessment in advance of a project commencing—policed by judicial review—can equally reduce long-term costs by refining decision making. Mitigative outcomes conducted in satisfaction of procedural requirements give effect to ideas of pluralist democracy,^183^ and arguably reallocate responsibility for costs from affected communities onto project developers (such as HS2 Limited), benefiting social welfare.^184^ A parallel could be drawn here with planning tools like the Community Infrastructure Levy or agreements under section 106 of the Town and Country Planning Act 1990, which both place obligations on developers that mitigate infrastructure’s social costs borne otherwise by communities.^185^ Perhaps, then, to frame citizen voice and legal risk as ‘raising costs’ is too simplistic, not fully capturing that costs, whether social or financial, are necessarily adopted by one actor or another. Whether procedural requirements, and the availability of litigation to enforce them, do in practice improve public administration is a question which may well differ case by case, and it is certainly arguable that defences of administrative law proceduralism are often made at too high a level of abstraction.^186^ This is an empirical question which judicial review scholars have largely shied away from, and a renewed empirical engagement with the granular impact of judicial review for good governance would be timely in the infrastructure field.^187^

Second, and looking beyond any individual project, legal actors increasingly recognise the economic importance of the rule of law.^188^ The UK has received criticism from the Aarhus Convention Compliance Committee for its access to environmental judicial review because, *inter alia*, features of the domestic Aarhus costs protection rules remain non-compliant with the Convention.^189^ At the same time, emphasising the economic gains of disincentivising infrastructure litigation, several think tanks have begun to propose constraining those same rules,^190^ or indeed withdrawing from the Convention entirely.^191^ Yet this overlooks the possible broader impact of restrictive reform on the UK’s international reputation as an investable state valuing the rule of law, which warrants further consideration. The government’s Banner Review, whose terms of reference emphasised complying with domestic and international legal obligations, wisely offered little support for such reforms while the UK remains a signatory to the Convention.^192^

Third, while the ‘chilling effect’ critique appropriately places litigation in its policy making context, it also risks isolating public law procedures from their social context. Where social opposition remains, constraining formal routes to express that opposition is unlikely to entirely minimise disruption to delivery. Notwithstanding its once near-unanimous support in the major political parties’ manifestos, HS2 has throughout its lifetime proven highly politically controversial, and brought some harms that were important to minimise. Mitigative concessions to allay citizens’ expressions of voice were therefore not necessarily due only to burdensome legal requirements. The much-criticised decision to construct the Chiltern Tunnel has been regarded as an expensive concession to appease Conservative voters’ political opposition^193^—an issue separate from the threat of litigation. This is a potential weakness of complaints about ‘citizen voice’: they risk conflating citizens’ expressions of voice made through straightforwardly political means, with litigious expressions. Indeed, this may represent an important distinction between these critiques and some classic ‘green light’ accounts of judicial scepticism. ‘Green light’ sceptics decry judicialisation of decision making and seek expanded political participation and internal routes of appeal. By contrast, in critiques of a chilling effect on infrastructure, participatory planning and judicial review, rather than representing alternative political and legal methods of public involvement, are mutually reinforcing. Judicial review represents an extension of a bloated planning system, exacerbating delays and giving teeth to legalistic procedural requirements. We therefore do not see criticism of judicial review that advocates for more expansive pre-decision participation processes enabling public involvement in appropriately political decision making, reducing the need for litigation. Rather, participatory planning is itself regarded as an inefficiency impeding (green) development, emblematic of a technocratic ‘impatience with process’.^194^ Yet attempts to streamline planning will be naturally contentious in developments which, like HS2, are highly politicised—precisely those developments where removing contention from one part of the system often leads democracies to address it in another (not least through litigation).^195^ Notably, the Banner Review indicated, as a potential cause of some judicial reviews, that some claimants feel disenfranchised by the streamlined DCO examination process.^196^ Similarly, judicial review is itself a feature of participative democracy—where it is restricted, more opponents may be incentivised to instead adopt protest tactics,^197^ a potentially more expensive and disruptive strategy which, as noted, has at points already plagued HS2’s construction process. As section 5 argued, judicial review in HS2 provided an official forum to air citizens’ concerns, but resolve them decisively and provide stability—thereby legally enforcing the project’s legitimacy amid social friction.^198^ One instrumental justification of public participation is that, where avenues of involvement are restricted, citizens may become alienated from authority.^199^ Accordingly, reducing the scope for litigation alongside other restrictions on participation could intensify disillusionment and strengthen a popular mandate for more disruptive direct action.

Fourth, ‘chilling effects’ on delivery are not isolated to litigation and participatory planning. It is strongly arguable that, more so than judicial review, HS2’s most prominent roadblocks have been central government indecision and a dearth of strategic vision for Britain’s transport network. Discussing the British political system’s seemingly unique capacity to deliver policy fiascos, Dunleavy has forcefully diagnosed the constitutional cause as the national executive’s unusual degree of centralised power, associated with ‘fastest law in the west’ policy making and limited internal checks on a small number of actors setting the national agenda.^200^ HS2 reflects quite how far the nation’s infrastructural future hinges on a few politicians, with infrastructure planning becoming more *ad hoc* and central government-dominated as the Coalition government moved away from long-term spatial strategic planning from 2010.^201^ George Osborne’s aforementioned commitment to a ‘northern powerhouse’ appears to have been a key driver in the Coalition government’s commitment to transport investment,^202^ seeking to deliver HS2 alongside Northern Powerhouse Rail. By contrast, and despite its repeated presence in manifestos and its parliamentary approval, ambivalence towards HS2 from a few key politicians amid rising costs in the years since has heightened uncertainty. With power so centralised in national government, those key politicians’ decisions carry enormous consequences. Taken on its own, the Johnson administration’s decision to commission the Oakervee Review in 2019, adding delay and uncertainty to the project’s future (not to mention spawning the *Packham* litigation), appeared unnecessary given the Review’s report recommended to deliver HS2 in full. Yet later political choices contradicted that report’s recommendations, with HS2 now stopping at Birmingham following the Johnson government’s 2021 Integrated Rail Plan and Rishi Sunak’s 2023 decision to cancel the route between Birmingham and Manchester. A future administration could reinstate HS2’s northern sections, yet that is somewhat beside the point. Where infrastructure policy is not articulated within a long-term strategic, geographic or temporal framework, it is unlikely to breed investors’ certainty that an area represents a stable, predictable environment for development.^203^ Indeed, any strategic infrastructural vision post-2010 has emerged away from central government, including through devolved organisations such as Transport for the North.^204^ Experience with transport delivery, and HS2 especially, sits uneasily with the depiction of those urging a release of central executive power to deliver infrastructure. Richard Johnson has argued that HS2’s high tunnelling costs demonstrate there is reason to be sceptical of the impact of devolving power, as tunnels were adopted through the Chiltern Hills in response to localised opposition.^205^ In arguing to strengthen central government’s role, though, Johnson obscures the costs and delay caused by that institution’s indecision. While that government indecision in part occurred in response to cost increases attributable to assertions of ‘citizen voice’, it has itself added costs and delay,^206^ and stopping HS2 at Birmingham undermines the project’s core benefits. Chilling effects also, then, result from the political sphere and the UK constitution’s characteristic centralisation. This should be a key concern for policy critics,^207^ and for research regarding public law’s role in facilitating or limiting economic growth.

## 7. Conclusion

Concern regarding the impact of liberal legalism on infrastructure delivery, amid climate and housing crises, perhaps represents the core emerging public law theme under the Labour government. This article has argued that the growing concern is worth taking seriously, and has engaged with two discourses—judicial power and litigant power—addressing judicial review’s role in infrastructure delivery, and policy implementation more broadly. Using HS2 as a case study, I have argued that the pattern of adjudication indicates an often marginal and minimalist judicial role which broadly legitimates government decision making and presents little concern regarding judicial power. Yet the article has also articulated an alternative (although, for judicial sceptics, potentially overlapping) critique of ‘litigant power’, which features less prominently in academic discussion but may gain influence under the Labour administration. This critique positions judicial review as compounding stifling layers of procedural regulation, thereby heightening project delays and chilling development through instilling overcaution, pre-emptive mitigation and uncertainty. Given its growing prominence and weighty concerns, this critique demands further scholarly attention, including through empirical ‘impact studies’ of judicial review. I have suggested, though, that while the HS2 experience indicates that litigant power is an important feature of the challenge in infrastructure delivery, it represents an oversimplistic and partial account.

The need for green infrastructure will likely see the Labour administration revisit attempts to streamline cumbersome infrastructure planning and litigation processes. Though well intentioned, such reform processes are cyclical, and have rarely quenched desires to make the conditions for infrastructure delivery more efficient and predictable. Importantly, focusing reformist attention on legalism risks overlooking the government’s role in shaping the appeal and stability of the development climate. I have suggested that critiques which focus on legalism’s ‘chilling effects’ but downplay the risk and chills resulting from the UK’s centralised decision-making structure are at best incomplete. Any suggestion that infrastructure policy would be more strategically delivered by further strengthening the national executive’s decisional role appears doubtful upon viewing successive governments’ indecisive backtracking in the HS2 saga, which has bred costs, delay and industry chilling effects. While the impacts on infrastructure delivery of litigant power deserve public lawyers’ empirical attention, so too do those of the UK’s centralised constitutional arrangement.

## Footnotes

1 Department for Levelling Up, Housing & Communities (DLUHC), *Getting Great Britain Building Again: Speeding Up Infrastructure Delivery* (2023) s 1.

2 ‘Keir Starmer’s Speech to the North East Chamber of Commerce’ (3 November 2023) <https://docs.google.com/document/d/1OknGy_tsh_7chGbSxbDvVX6agokzvTFu6etjEKj3xXw/edit>. All URLs in this article last accessed 10 March 2025.

3 Lord Banner KC, ‘Independent Review into Legal Challenges against Nationally Significant Infrastructure Projects’ (Ministry of Housing, Communities and Local Government, 28 October 2024).

4 HC Deb 23 January 2025, vol 760, col 37WS.

5 Joe Tomlinson and Simon Halliday, ‘Does Administrative Law Inhibit Good Government?’ (2024) 28(2) Edinburgh Law Review 264.

6 eg Ministry of Justice, *The Independent Review of Administrative Law* (Cm 407, 2021); Richard Ekins, *Protecting the Constitution: How and Why Parliament Should Limit Judicial Power* (Policy Exchange 2019).

7 Britain Remade, ‘About’ <www.britainremade.co.uk/about>. See eg Sam Dumitriu, *Powerbook: A Playbook for Energy Security by 2030* (Britain Remade 2023); Gabriel Moberg and David Lawrence, ‘Reforming Judicial Review to Get Britain Building’ (UKDayOne, 16 October 2024) <https://ukdayone.org/briefings/reforming-judicial-review-to-get-britain-building>.

8 See Zachary Liscow, ‘Getting Infrastructure Built: The Law and Economics of Permitting’ (2025) 39(1) Journal of Economic Perspectives 151, 175–6.

9 See Ekins (n 6).

10 eg Joanna Bell and Elizabeth Fisher, ‘Exploring a Year of Administrative Law Adjudication in the Administrative Court’ [2021] PL 505; Richard Kirkham and Elizabeth A O’Loughlin, ‘Judicial Review and Ombuds: A Systematic Analysis’ [2020] PL 680.

11 Banner (n 3).

12 DLUHC (n 1) s 1.

13 Maurice Sunkin, ‘What Is Happening to Applications for Judicial Review?’ (1987) 50(4) MLR 432, 460.

14 AP Le Sueur and Maurice Sunkin, ‘Applications for Judicial Review: The Requirement of Leave’ [1992] PL 102, 104–5.

15
*Review of the Crown Office List* (Lord Chancellor’s Department 2000).

16 Michael Fordham KC and others, ‘Streamlining Judicial Review in a Manner Consistent with the Rule of Law’ (Bingham Centre for the Rule of Law 2014) Bingham Centre Report 2014/01, 4; Catherine Haddon, Raphael Hogarth and Alex Nice, *Judicial Review and Policy Making: The Role of Legal Advice in Government* (Institute for Government 2021) 10.

17 Matthew White, ‘Attaining the Age of Consents: Five Years of the Planning Act 2008’ [2013] JPL OP100, OP147.

18 Banner (n 3) para 50.

19 ibid paras 96, 110.

20 Leah Brooks and Zachary Liscow, ‘Infrastructure Costs’ (2023) 15(2) American Economic Journal: Applied Economics 1, 22.

21 ibid 15, 24.

22 ibid 26.

23 Charles R Epp, ‘The Judge Over Your Shoulder: Is Adversarial Legalism Exceptionally American?’ (2003) 28(3) L & Soc Inquiry 743, 754.

24 See Sam Guy, ‘Mobilising the Market: An Empirical Analysis of Crowdfunding for Judicial Review Litigation’ (2023) 86(2) MLR 331.

25 ibid 339–40; Carolyn Abbot, ‘Losing the local? Public Participation and Legal Expertise in Planning Law’ (2020) 40(2) LS 269.

26 Sam Dumitriu, ‘Building Back Faster’ (*Works in Progress*, 24 February 2023) <https://worksinprogress.co/issue/building-back-faster/>. See also Liscow (n 8) 156.

27 Tommaso Pavone and Øyvind Stiansen, ‘The Shadow Effect of Courts: Judicial Review and the Politics of Preemptive Reform’ (2022) 116(1) American Political Science Review 322, 322, 334.

28 Robert A Kagan, ‘Adversarial Legalism and American Government’ (1991) 10(3) Journal of Policy Analysis and Management 369, 378, 386.

29 ibid 377.

30 James W Coleman, ‘Pipelines & Power-Lines: Building the Energy Transport Future’ (2019) 80(2) Ohio St LJ 263, 293–4.

31 Dumitriu, ‘Building Back Faster’ (n 26).

32 Dominic Cummings, ‘The Hollow Men II: Some Reflections on Westminster and Whitehall Dysfunction’ (*Dominic Cummings’s Blog*, 30 October 2014) <https://dominiccummings.com/2014/10/30/the-hollow-men-ii-some-reflections-on-westminster-and-whitehall-dysfunction/>.

33 Government Legal Department, *The Judge Over Your Shoulder* (6th edn, 2022).

34 Haddon, Hogarth and Nice (n 16) 9.

35 For discussion, see Tomlinson and Halliday (n 5).

36 Ministry of Justice, *Judicial Review: Proposals for Further Reform* (Cm 8703, 2013) para 7.

37
*Independent Review of Administrative Law* (Ministry of Justice) <www.gov.uk/government/groups/independent-review-of-administrative-law>.

38 Mark Elliott, ‘Constitutional Adjudication and Constitutional Politics in the United Kingdom: The *Miller II* Case in Legal and Political Context’ (2020) 16 EuConst 625, 644.

39 ibid 628–9.

40 eg Richard Ekins, *Brexit and Judicial Power* (Policy Exchange 2016).

41 eg Sir Stephen Laws KCB, *How to Address the Breakdown of Trust Between Government and Courts* (Policy Exchange 2021) 17–18; Richard Ekins and Julie Marionneau, *Lawfare: Resisting the Judicialisation of War* (Policy Exchange 2019).

42 Richard Ekins and Graham Gee, ‘*Miller*, Constitutional Realism and the Politics of Brexit’ in Mark Elliott, Jack Williams and Alison Young (eds), *The UK Constitution after Miller: Brexit and Beyond* (Hart Publishing 2018).

43 Judicial Power Project, ‘About the Judicial Power Project’ <https://judicialpowerproject.org.uk/about/>.

44 Rt Hon Lord Hermer KC, ‘The Rule of Law in an Age of Populism’ (Bingham Centre for the Rule of Law 15 October 2024) <www.gov.uk/government/speeches/attorney-generals-2024-bingham-lecture-on-the-rule-of-law>.

45 HC Deb 23 January 2025 (n 4).

46 Dumitriu, *Powerbook* (n 7) 6.

47 Samuel Hughes, *Accelerating Infrastructure: How to get Britain building more, faster* (Centre for Policy Studies 2024).

48 Moberg and Lawrence (n 7).

49 Sam Dumitriu, ‘Will Labour Actually Get Britain Building Again?’ (Notes on Growth 28 May 2024) <www.samdumitriu.com/p/will-labour-actually-get-britain>.

50 CPR 46.24. See Carol Day and others, *A Pillar of Justice II* (ELF, FoE, RSPB 2023).

51 Dumitriu, ‘Will Labour’ (n 49).

52 Banner (n 3).

53 Carol Harlow and Richard Rawlings, ‘“Striking Back” and “Clamping Down”: An Alternative Perspective on Judicial Review’ in John Bell and others (eds), *Public Law Adjudication in Common Law Systems: Process and Substance* (Hart Publishing 2016).

54 eg Moberg and Lawrence (n 7); Kane Emerson and Samuel Hughes, ‘New Towns for a New Britain: Where Britain’s Next New Town Should Be’ (UKDayOne 15 July 2024) <https://ukdayone.org/briefings/new-towns-for-a-new-britain-where-britain-s-next-new-town-should-be>.

55 Department for Transport, *High Speed Two: An Engine for Growth* (11 September 2013).

56 Department for Transport, *The Strategic Case for HS2* (October 2013) 12.

57 ibid 71.

58 See Douglas Oakervee, *Oakervee Review* (Department for Transport and HS2 Limited 2019).

59 Department for Transport, *The Strategic Case for HS2* (n 56) 81–2.

60 Daniel Straulino and others, ‘Connecting Up Embedded Knowledge across Northern Powerhouse Cities’ (2023) 55(7) Environment and Planning A: Economy and Space 1690, 1693.

61 See Jona Razzaque and Claire Lester, ‘Why Protect Ancient Woodland in the UK? Rethinking the Ecosystem Approach’ (2021) 10(1) TEL 135.

62 Department for Transport and High Speed 2 Limited, ‘HS2 Phase One Environmental Statement: Documents’ <www.gov.uk/government/collections/hs2-phase-one-environmental-statement-documents>.

63 eg Philip Georgiadis, Jim Pickard and Gill Plimmer, ‘HS2 Spent £100mn on Tunnel to Protect Rare Bats’ *Financial Times* (London, 7 November 2024) <www.ft.com/content/fd5e34dc-e006-491b-93b2-576e3adf45f8>.

64 National Audit Office, *High Speed Two: A Progress Update* (2019–20, HC 40) 41.

65 Mariana Valverde, *Infrastructure: New Trajectories in Law* (Routledge 2022) 76; Richard Johnson, ‘The Limits of Devolution for the Left’ (2024) 31(1) IPPR Progressive Review 50.

66 Oakervee (n 58).

67 Department for Transport, *Integrated Rail Plan for the North and Midlands* (18 November 2021).

68 Jim Pickard and Peter Campbell, ‘Rishi Sunak to Tout Pro-car Agenda in Tory Conference Pitch to Voters’ *Financial Times* (London, 28 September 2023) <www.ft.com/content/c09f7bca-ed99-4e8c-9a7e-7229023293cd>.

69 Carol Harlow and Richard Rawlings, *Law and Administration* (4th edn, CUP 2021) 632, 636.

70 eg Department of the Environment, Transport and the Regions, ‘Modernising Planning: Streamlining the Processing of Major Projects through the Planning System (Consultation Paper)’ (1999) JPL B120, B121.

71 John Popham and Michael Purdue, ‘The Future of the Major Inquiry’ [2002] JPL 137, 138; Timothy O’Riordan, Ray Kemp and Michael Purdue, *Sizewell B: An Anatomy of the Inquiry* (Macmillan Press 1988). See also discussion in *Bushell v Secretary of State for the Environment* [1980] UKHL 1.

72 Victoria Jenkins, ‘Planning for Major Infrastructure in England: Front-Loading Participation in the Interests of Efficiency’ in Chris Backes, Mariolina Eliantonio and Sander Jansen (eds), *Quality and Speed in Administrative Decision-making: Tension or Balance?* (Intersentia 2016).

73 Popham and Purdue (n 71) 144–5; William Walton, ‘The Future of Major Public Planning Inquiries in Britain’ (2001) 72(4) Town Planning Review. See also some citizens’ perceptions of the Channel Tunnel’s approval: Eve Darian-Smith, *Bridging Divides: The Channel Tunnel and English Legal Identity in the New Europe* (University of California Press 1999) 121–5.

74
*R (HS2 Action Alliance Ltd) v Secretary of State for Transport and another* [2014] UKSC 3 (*HS2AA 1* UKSC).

75 Mark Laurence Wilde, ‘All the Queen’s Horses: Statutory Authority and HS2’ (2017) 37(4) LS 765, 765. See also Matthew England and Ruth Fox, ‘HS2 Fiasco: What Does It Mean for Parliament?’ (Hansard Society 15 October 2023) <www.hansardsociety.org.uk/blog/hs2-fiasco-what-does-it-mean-for-parliament>.

76 White (n 17) OP142.

77 Sam Watling and Matilda Davies, ‘From HS2 to Wembley, Why Can’t Britain Build on Budget?’ *The Times* (London, 29 October 2023) <www.thetimes.co.uk/article/from-hs2-to-wembley-why-cant-britain-build-on-budget-9k6xgs8c6>.

78 Emerson and Hughes (n 54).

79 Britain Remade and Create Streets, *Creating New Towns Fast and Well* (2024) 9.

80 The keyword search was: “hs2 OR high-speed two OR high speed two OR high-speed 2 OR high speed 2”.

81
*Rukin v Secretary of State for Transport* CO/2470/2020; *R (Packham) v Secretary of State for Transport and the Prime Minister* [2020] EWCA Civ 1004 (*Packham* EWCA); *R (Keir) v Natural England* [2021] EWHC 1059 (Admin); *R (Misbourne Environmental Protection Ltd) v Environment Agency* [2021] EWHC 3094 (Admin); *Siemens Mobility Ltd v HS2 Limited* [2023] EWHC 2768 (TCC).

82
*R (Maxey) v HS2 Limited* [2021] EWHC 246 (Admin).

83
*HS2AA 1* UKSC (n 74); *R (HS2 Action Alliance Ltd and anor) v Secretary of State for Transport* [2014] EWCA Civ 1578 (*HS2AA 2* EWCA); *R (Granger-Taylor) v HS2 Limited* [2020] EWHC 1442 (Admin); *R (London Borough of Hillingdon Council) v Secretary of State for Transport and Secretary of State for Housing, Communities and Local Government* [2021] EWCA Civ 1501 (*Hillingdon 2* EWCA); *Buckinghamshire Council v Secretary of State for Transport and Secretary of State for Levelling Up, Housing and Communities* [2022] EWHC 1923 (Admin).

84
*HS2AA 1* UKSC (n 74). Its constitutional importance lies in its treatment of the relationship between EU law and domestic constitutional principles.

85
*R (London Borough of Hillingdon) v Secretary of Transport and Secretary of State for Housing, Communities and Local Government* [2020] EWCA Civ 1005 (*Hillingdon 1* EWCA).

86
*Patentes Talgo SL v High Speed Two Ltd* [2021] EWHC 3780 (TCC).

87
*R (Thornton) v Secretary of State for Transport and R (Information Commissioner) v Secretary of State for Transport and ors* [2014] EWHC 2700 (Admin).

88 Julian Milford, ‘High Speed Trains and Black Spider Letters: Freedom of Information and the Ministerial Veto’ (2015) 20(4) JR 206, 214.

89
*HS2AA 2* EWCA (n 83).

90
*Packham v Secretary of State for Transport and the Prime Minister* [2020] EWHC 829 (Admin) (*Packham* EWHC).

91
*Packham* EWCA (n 81).

92
*Rukin* (n 81).

93
*Patentes Talgo* (n 86); Catherine Moore, ‘HS2 Reaches Out of Court Settlement with Talgo in Train Procurement Dispute’ *New Civil Engineer* (London, 28 June 2021) <www.newcivilengineer.com/latest/hs2-reaches-out-of-court-settlement-with-talgo-in-train-procurement-dispute-28-06-2021/>.

94
*Siemens* (n 81).

95 ibid [826]–[828].

96
*Keir* (n 81); *Misbourne EP* (n 81); *Packham* EWCA (n 81); *Rukin* (n 81); *Maxey* (n 82); *Thornton* (n 87); *HS2AA 1* UKSC (n 74); *HS2AA 2* EWCA (n 83).

97
*Keir* (n 81).

98 Reg 55.

99
*Keir* (n 81) [69]–[71].

100 ibid [109].

101 HS2, ‘Chiltern Tunnel’ <www.hs2.org.uk/building-hs2/tunnels/tunnel-drives/chiltern-tunnel/>.

102
*Misbourne EP* (n 81) [21]–[23].

103 Dr Paul Thornton, ‘Exact Route Details Revealed of HS2 Route via Derby’ (StopHS2 7 October 2013) <https://stophs2.org/news/9497-exact-hs2-route-derby>; High Speed Rail (London—West Midlands) Bill Select Committee, *Petitions against the High Speed Rail (London—West Midlands) Bill* (HC 2014–15).

104 Yseult Marique and Steven Van Garsse, ‘Public–Private Cooperation and Judicial Review: A Case Study Drawn from European Infrastructure Projects’ (2018) 24(3) EPL 515, 527.

105
*R (Buckinghamshire County Council and ors) v Secretary of State for Transport* [2013] EWHC 481 (Admin) (*HS2AA 1* EWHC).

106 eg *Hillingdon Borough Council v Persons Unknown and ors* [2020] EWHC 2153 (QB); *HS2 Limited and anor v Four Categories of Persons Unknown and ors* [2022] EWHC 2360 (KB); *H2S Limited and anor v Persons Unknown and ors* [2024] EWHC 1277 (KB).

107 Carol Harlow and Richard Rawlings, *Pressure Through Law* (Routledge 1992) 7.

108
*Maxey* (n 82).

109
*HS2 Limited v Maxey and ors* [2022] EWHC 1010 (QB).

110
*HS2AA 1* EWHC (n 105); *HS2AA 2* EWCA (n 83); *Hillingdon 1* EWCA (n 85); *Hillingdon 2* EWCA (n 83); *Buckinghamshire* (n 83).

111 Per appeal rights in the 2017 Act, sch 17, para 22.

112
*R (London Borough of Hillingdon) v Secretary of Transport and Secretary of State for Housing, Communities and Local Government* [2019] EWHC 3574 (Admin), [77], [85].

113
*Hillingdon 1* EWCA (n 85) [68].

114 ibid [77].

115 ibid [70].

116
*London Borough of Hillingdon v Secretary of State for Transport and Secretary of State for Housing, Communities and Local Government* [2021] EWHC 871 (Admin) (*Hillingdon 2* EWHC).

117
*Hillingdon 2* EWCA (n 83).

118
*Hillingdon 2* EWHC (n 116) [208]–[209].

119
*Hillingdon 2* EWCA (n 83) [57].

120
*Buckinghamshire* (n 83).

121 Kagan (n 28) 377.

122 Bell and Fisher (n 10); Kirkham and O’Loughlin (n 10).

123
*Keir* (n 81) [115].

124
*Packham* EWHC (n 90) [73].

125 ibid [133].

126 On which, see Joanna Bell, *The Anatomy of Administrative Law* (Hart Publishing 2020); Sarah Nason, *Reconstructing Judicial Review* (Hart Publishing 2016).

127 Richard Macrory and Michael Woods, *Modernizing Environmental Justice* (Centre for Law and the Environment, UCL 2003) 21–2; Ole W Pedersen, ‘A Study of Administrative Environmental Decision-Making before the Courts’ (2019) 31(1) JEL 59, 68–9.

128 For similar discussion, see Sanja Bogojević, ‘Squaring the Circle? Regional Airport Expansion, Climate Change and the Planning Regime’ (2024) 87(4) MLR 967.

129
*Hillingdon 2* EWCA (n 83).

130
*Keir* (n 81).

131
*Packham* EWCA (n 81); *HS2AA 1* EWHC (n 105).

132
*Keir* (n 81) [44].

133
*Misbourne EP* (n 81) [29]–[30].

134
*Granger-Taylor* (n 83) [97].

135 ibid [117].

136
*Maxey* (n 82) [26].

137 W Ivor Jennings, ‘Courts and Administrative Law—the Experience of English Housing Legislation’ (1936) 49(3) Harv L Rev 426.

138 See recently Sanjit Nagi, ‘A Future Constitutional Battleground? Expropriation, Compensation, and the Right to Peaceful Enjoyment of Property under the European Convention on Human Rights’ (UK Constitutional Law Association 15 April 2024) <https://ukconstitutionallaw.org/2024/04/15/sanjit-nagi-a-future-constitutional-battleground-expropriation-compensation-and-the-right-to-peaceful-enjoyment-of-property-under-the-european-convention-on-human-rights/>.

139
*HS2AA 1* EWHC (n 105) [468], [480].

140
*R (HS2 Action Alliance Ltd and anor) v Secretary of State for Transport* [2014] EWHC 2759 (Admin) (*HS2AA 2* EWHC); *HS2AA 2* EWCA (n 83).

141
*Granger-Taylor* (n 83) [119]–[120]. See *Thomas and ors v Bridgend CBC* [2011] EWCA Civ 862.

142 Kirkham and O’Loughlin (n 10) 696.

143 TT Arvind, Simon Halliday and Lindsay Stirton, ‘Judicial Review and Administrative Justice’ in Marc Hertogh and others (eds), *The Oxford Handbook of Administrative Justice* (OUP 2021) 80.

144 Emerson and Hughes (n 54).

145 Moberg and Lawrence (n 7).

146 See *Granger-Taylor* (n 83); *HS2AA 2* EWHC (n 140).

147
*Packham* EWCA (n 81).

148
*Keir* (n 81) [114], [120].

149 Banner (n 3) para 50.

150
*Packham* EWHC (n 90) [122], [125].

151 ibid [42].

152 ibid [35].

153 Lee Marsons, ‘Crossing the t’s and Dotting the i’s: The Turn to Procedural Rigour in Judicial Review’ [2023] PL 29.

154 Moberg and Lawrence (n 7); Ministry of Justice, *Judicial Review* (n 36) paras 6–8.

155 See Maurice Sunkin and Varda Bondy, ‘The Use and Effects of Judicial Review: Assumptions and the Empirical Evidence’ in Bell and others (n 53) 331–2.

156 Liscow (n 8) 153.

157 Ministry of Justice, *Judicial Review* (n 36) para 47.

158 David Elvin KC, ‘The Planning Court’ (2014) 19(2) JR 98, 98. See *London & Henley Ltd (and ors) v Secretary of State for Communities and Local Government (and ors)* [2014] EWHC 4207 (Admin).

159 CPR, Part 54.21, PD 54D. See Elvin (n 158).

160 PD 54D, III.

161 Mr Justice Lindblom, ‘The Planning Court: One Year On’ [2015] JPL OP3.

162 Ministry of Justice, *Civil Justice and Judicial Review Data* <www.gov.uk/government/statistics/civil-justice-statistics-quarterly-april-to-june-2024>.

163 Banner (n 3) paras 38–42.

164 Jenkins (n 72) 107.

165 Angus Walker, ‘Comment—Court Reduces Planning Case Time’ *Planning* (7 November 2014).

166 Banner (n 3) paras 112–13.

167 Fordham and others (n 16).

168 ibid 21; Banner (n 3) para 76.

169 Banner (n 3).

170 See Sam Guy, ‘The Government’s Plan to Reform Infrastructure Judicial Review’ (UK Constitutional Law Association 28 January 2025) <https://ukconstitutionallaw.org/2025/01/28/sam-guy-the-governments-plan-to-reform-infrastructure-judicial-review/>.

171 Tim Marshall and Richard Cowell, ‘Infrastructure, Planning and the Command of Time’ (2016) 34(8) Environment and Planning C: Politics and Space 1843, 1843.

172 DLUHC (n 1) s 1. See also Ministry of Justice, *Judicial Review* (n 36) para 36.

173 Dumitriu, ‘Building Back Faster’ (n 26).

174 Wilde (n 75) 774–5.

175 ibid 766.

176 ibid 780.

177 Valverde (n 65) 76.

178
*Misbourne EP* (n 81).

179 Notably, the group crowdfunded this claim.

180 See DJ Galligan, *Due Process and Fair Procedures: A Study of Administrative Procedures* (OUP 1996) 131–2.

181 Georgiadis, Pickard and Plimmer (n 63).

182
*Keir* (n 81).

183 Kagan (n 28) 384.

184 Brooks and Liscow (n 20) 25.

185 Edward Mitchell, ‘Contracting in the Public Interest? Re-examining the Role of Planning Obligations in Contemporary Town Planning Processes’ (2024) 77(1) CLP 259; Tola Amodu, ‘Revisiting the Rules. The Pervasiveness of Discretion in the Context of Planning Gains: The Case of the Community Infrastructure Levy’ [2020] PL 643.

186 Nicholas Bagley, ‘The Procedure Fetish’ (2019) 118(3) Mich L Rev 345, 369.

187 See Genevra Richardson, ‘Impact Studies in the UK’ in Marc Hertogh and Simon Halliday (eds), *Judicial Review and Bureaucratic Impact: International and Interdisciplinary Perspectives* (CUP 2004).

188 Lord Hodge, ‘The Rule of Law, the Courts and the British Economy’ (Guildhall Lecture, 4 October 2022) <https://supremecourt.uk/uploads/the_rule_of_law_the_courts_and_the_british_economy_54eaa5ca0a.pdf>; Murray Hunt, ‘Restoring the UK’s Economic Credibility Requires Rule of Law Leadership’ (Bingham Centre for the Rule of Law 17 November 2022) <https://binghamcentre.biicl.org/comments/125/restoring-the-uks-economic-credibility-requires-rule-of-law-leadership?cookiesset=1&ts=1670344674>.

189 UNECE, *Decision VII/8s concerning compliance by the United Kingdom of Great Britain and Northern Ireland with its obligations under the Convention*.

190 Dumitriu, ‘Will Labour’ (n 49); Moberg and Lawrence (n 7).

191 Hughes (n 47).

192 Banner (n 3) para 60.

193 Valverde (n 65) 76; Johnson (n 65).

194 Chiara Armeni and Maria Lee, ‘Participation in a Time of Climate Crisis’ (2021) 48(4) J L Soc’y 549, 560.

195 Marshall and Cowell (n 171) 1859–60.

196 Banner (n 3) para 31.

197 See Chris Hilson, ‘New Social Movements: The Role of Legal Opportunity’ (2002) 9(2) Journal of Economic Perspectives 238, 250.

198 Sanja Bogojević and Mimi Zou, ‘Making Infrastructure “Visible” in Environmental Law: The Belt and Road Initiative and Climate Change Friction’ (2021) 10(1) TEL 35, 36, 42.

199 Skeffington Committee, *People and Planning: Report of the Committee on Public Participation in Planning* (HMSO 1969) para 8.

200 Patrick Dunleavy, ‘Policy Disasters: Explaining the UK’s Record’ (1995) 10(2) Public Policy and Administration 52.

201 Tim Marshall, ‘Infrastructure Planning and Spatial Planning: Current Relationships in the UK’ (2020) 91(1) Town Planning Review 47, 65.

202 Iain Docherty and others, ‘The Curious Death—and Life?—of British Transport Policy’ (2018) 36(8) Environment and Planning C: Politics and Space 1458, 1465.

203 Martin Boddy and Hannah Hickman, ‘The Demise of Strategic Planning? The Impact of the Abolition of Regional Spatial Strategy in a Growth Region’ (2013) 84(6) Town Planning Review 743, 753, 757.

204 Marshall (n 201) 54, 58.

205 Johnson (n 65).

206 Jessica Elgot, ‘Internal Government Briefing Admits HS2 Delays Will Increase Costs’ *The Guardian* (London, 14 March 2023) <www.theguardian.com/uk-news/2023/mar/14/internal-government-briefing-hs2-delays-increase-costs>.

207 Britain Remade do recognise this: Sam Dumitriu and others, *Back on Track* (Britain Remade and Create Streets 2024).

